# Comparison of SP263 and 22C3 pharmDx assays to test programmed death ligand‐1 (PD‐L1) expression in surgically resected non‐small cell lung cancer

**DOI:** 10.1111/1759-7714.15319

**Published:** 2024-05-03

**Authors:** Naoko Shigeta, Shuji Murakami, Tomoyuki Yokose, Tetsuya Isaka, Kanako Shinada, Takuya Nagashima, Hiroyuki Adachi, Shunsuke Shigefuku, Kotaro Murakami, Jun Miura, Noritake Kikunishi, Kozue Watabe, Haruhiro Saito, Hiroyuki Ito

**Affiliations:** ^1^ Department of Thoracic Surgery Kanagawa Cancer Center Yokohama Japan; ^2^ Department of Thoracic Oncology Kanagawa Cancer Center Yokohama Japan; ^3^ Department of Pathology Kanagawa Cancer Center Yokohama Japan

**Keywords:** immune checkpoint inhibitors (ICIs), non‐small cell lung cancer (NSCLC), PD‐L1 immunohistochemistry assay, programmed death ligand‐1

## Abstract

**Background:**

Atezolizumab, one of the immune checkpoint inhibitors, has been approved as an adjuvant treatment following resection and platinum‐based chemotherapy in patients with stage II–IIIA non‐small cell lung cancer with 1% or more programmed death ligand‐1 (PD‐L1) expression. The Food and Drug Administration (FDA) has approved SP263 as a companion diagnostic assay for adjuvant treatment with atezolizumab; however, in clinical practice, the 22C3 assay is most commonly used for advanced non‐small cell lung cancer. Therefore, our study aimed to compare two PD‐L1 assays, SP263 and 22C3, to evaluate whether 22C3 could replace SP263 when deciding whether to administer adjuvant atezolizumab.

**Methods:**

We retrospectively and prospectively analyzed 98 patients who underwent surgical resection at Kanagawa Cancer Center (Japan). An immunohistochemistry assay was performed for all the cases with both SP263 and 22C3. We statistically analyzed the concordance of PD‐L1 expression between SP263 and 22C3 assays.

**Results:**

The concordance between the two assays using Cohen's kappa was *κ* = 0.670 (95% CI: 0.522–0.818) at the 1% cutoff and *κ* = 0.796 (95% CI: 0.639–0.954) at the 50% cutoff. The Spearman correlation coefficient of 0.874 (*p* < 0.01) indicated high concordance. PD‐L1 expression with 22C3 resulted slightly higher than that with SP263.

**Conclusions:**

This study showed a high concordance of PD‐L1 expression with the SP263 and 22C3 assays. Further studies examining the therapeutic effects of adjuvant atezolizumab are required.

## Graphical abstract

This study showed a high concordance of programmed death ligand‐1 (PD‐L1) expression with the SP263 and 22C3 assays, with the 22C3 assay having a slightly higher frequency of positivity. These results suggest that the 22C3 assay could be used instead of the SP263 assay to determine whether to administer adjuvant atezolizumab.

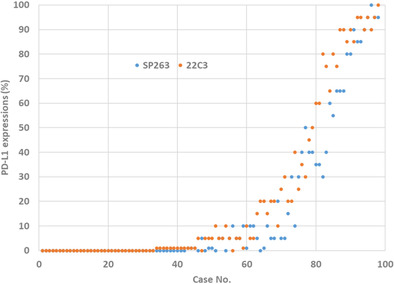

## INTRODUCTION

Immune checkpoint inhibitors (ICIs) such as programmed death‐1/programmed death ligand‐1 (PD‐1/PD‐L1) inhibitors have shown remarkable clinical benefits in the treatment of metastatic and locally advanced non‐small cell lung cancer (NSCLC).^1^, ^2^, ^3^, ^4^, ^5^ PD‐L1 expression in tumor cells using immunohistochemistry (IHC) assay is a biomarker for predicting the efficacy of PD‐1/PD‐L1 inhibitor.^6^ Clinical trials have been conducted using different PD‐L1 IHC assays for each PD‐1/PD‐L1 inhibitor, and the results have established the effectiveness of the treatment, providing clear evidence of its therapeutic efficacy. Therefore, each PD‐1/PD‐L1 inhibitor has a different companion diagnostic: 28‐8 (Dako) with nivolumab (Bristol‐Myers Squibb), 22C3 (Dako) with pembrolizumab (Merck & Co., Inc.), SP263 (Ventana) with durvalumab (AstraZeneca), and SP142 (Ventana) with atezolizumab (Genentech).^7^ Based on the results of a phase III study (KEYNOTE‐024), in which pembrolizumab significantly improved overall survival (OS) and progression‐free survival (PFS) compared to platinum‐based chemotherapy in metastatic NSCLC patients with 50% or more PD‐L1 tumor proportion score (TPS) assessed using the 22C3 (Dako) IHC assay^8^; in clinical practice, the 22C3 assay is the most commonly used assay for advanced NSCLC.

Atezolizumab is one of the PD‐L1 inhibitors that have been approved for the first‐line treatment of metastatic NSCLC with high PD‐L1 and second‐line treatment of metastatic NSCLC with any PD‐L1 expression. IMpower010, a randomized, multicenter, open label phase III trial demonstrated that adjuvant atezolizumab treatment improved disease‐free survival compared with the best supportive care in patients with resected stage II‐IIIA NCSLC with 1% or more PD‐L1 expression (hazard ratio [HR] 0.66, 95% confidence interval (CI): 0.50–0.88; *p* = 0.0039).^9^ Based on these results, atezolizumab has been approved for adjuvant treatment following resection and platinum‐based chemotherapy in patients with stage II–IIIA NSCLC with 1% or more PD‐L1 expression. In the IMpower010 trial, the hazard ratio (HR) for disease‐free survival in NSCLC patients with PD‐L1 expression of 1%–49% and more than 50% were 0.87 (95% CI: 0.60–1.26) and 0.43 (95% CI: 0.27–0.68), respectively. HR for OS with PD‐L1 expression of 1%–49% and more than 50% were 1.22 (95% CI: 0.71–2.10) and 0.37 (95% CI: 0.18–0.74), respectively. Therefore, the Japanese clinical guideline recommended adjuvant treatment with atezolizumab for NSCLC with 50% or more PD‐L1 expression; however, for 1%–49%, the guideline stated that the evidence was not clear enough to recommend it. Whether PD‐L1 expression is <1%, 1%–49% or ≧50% is extremely crucial to know to maximize the benefit of PD‐L1 inhibitors with immune‐related adverse events.

Because PD‐L1 expression levels influence the eligibility for treatment with ICIs, PD‐L1 assays need to be reliable and accurate. In the IMpower010 trial, PD‐L1 expression was evaluated using an IHC assay of SP263, and the FDA approved SP263 as a companion diagnostic of adjuvant treatment with atezolizumab. There have been reports of high concordance and discordance among PD‐L1 assays. The Blueprint PD‐L1 IHC assay comparison project, which included 81 lung cancer specimens of various sample types, such as resections, core needle or bronchial biopsy samples, tumor‐positive lymph node samples, and cytological cell blocks, concluded that 22C3 and SP263 were comparable. However, the results showed that SP263 had a slightly higher frequency of positivity than 22C3.^10^ Based on this result, some cases may have been missed in the 22C3 assay when determining whether to administer adjuvant atezolizumab. Thus, our study aimed to compare two PD‐L1 assays, SP263 and 22C3 in a large series of real‐world lung cancers using only surgically complete resection specimens and to evaluate whether 22C3 can replace SP263 when deciding whether to administer adjuvant atezolizumab.

## METHODS

### Study cohort

We retrospectively collected data from 48 patients who underwent surgery between May 2022 and October 2022 and prospectively collected data from 54 patients who underwent surgery after the period for which data were collected retrospectively. We consecutively evaluated PD‐L1 expression in the retrospective and prospective cases. Finally, 98 patients who underwent surgery between May 2022 and February 2023 were included. Patient data were obtained from electronic medical records. Ethical approval for this study was obtained from the Kanagawa Cancer Center (no. 2022‐64). Informed consent was obtained from all the patients.

### Inclusion and exclusion criteria

Patients who underwent surgical resection at the Kanagawa Cancer Center (Japan) and who were pathologically diagnosed with stage IB‐III NSCLC were included in this study. Patients who refused to be evaluated for PD‐L1 expression and patients who received any preoperative treatment were excluded.

### Immunohistochemical analysis

Immunohistochemistry was performed on primary lesions of surgically resected NSCLC using formalin‐fixed tissue sections. In all cases, the same formalin‐fixed tissue blocks were used for SP263 and 22C3 assays. Formalin‐fixed, paraffin‐embedded tissue blocks were cut into serial 4‐μm‐thick sections and deparaffinized. Immunostaining using clone SP263 (Roche Diagnostics) was performed with a Ventana BenchMark platform (Ventana Medical Systems) and 22C3 pharmDx (Dako, Agilent Technologies) was performed using a Dako Autostainer Link 48 platform. Each case was stained with both SP263 and 22C3 assays, the protocols of which are detailed in the product inserts and autostainers. In both assays, lung tissues confirmed positive and negative for PD‐L1 are generally stained at the same time as the specimens and for the SP263 assay, in addition, placental tissue is stained at the same time. It was conducted at SRL, Japan's largest company in the clinical testing business, which is entrusted with specimens from medical institutions and delivers the test results. A trained pathologist evaluated the tumor proportion score (TPS) for each case after negative control specimens confirmed no positive findings. The immunohistochemistry procedure was the same for all cases. Using the cutoff values commonly used in clinical practice and clinical trials, we defined PD‐L1 expression of <1% as negative, 1%–49% as weakly positive, and ≧50% as strongly positive.

### Statistical analysis

To compare the clinical performance of the assays, overall percent agreement (OPA), positive percent agreement (PPA), negative percent agreement (NPA), and Cohen's *κ* at each cutoff value (≥1% and ≥50%) were calculated. Scores of *κ* values less than 0.60 as indicating weak, 0.60–0.79 as moderate, 0.80–0.90 as strong, and above 0.90 as almost perfect agreement.^11^ The intraclass correlation coefficient (ICC) was used to assess scoring reliability across the two assays for continuous TPS scores of 0%–100%. The values less than 0.5, between 0.5 and 0.75, between 0.75 and 0.9, and greater than 0.9, respectively, indicated poor, moderate, good, and excellent reliability.^12^ Spearman's rank correlation coefficient was used to analyze the relationship between the two assays. The range is −1 to +1, where 0 indicates no correlation, and the correlation becomes stronger as the absolute value of the coefficient approaches 1.^13^ All statistical analyses were performed using EZR (Saitama Medical Center, Jichi Medical University, Saitama, Japan), which is a graphical user interface for R (R Foundation for Statistical Computing, Vienna, Austria).^14^

## RESULTS

### Patient and tumor characteristics

A total of 98 patients were included in this analysis. The clinicopathological features of the patients are summarized in Table 1. The median patient age was 72.5 (42–89) years, and 64 males and 34 females were included. The histological subtypes included 62 adenocarcinomas, 27 squamous cell carcinomas, one adenosquamous cell carcinoma, seven large cell carcinomas, and one carcinosarcoma. The pathological stages were as follows: 30 cases were IB, 11 were IIA; 29 were IIB, 18 were IIIA, and 10 were IIIB.

**TABLE 1:** Patient characteristics for 98 NSCLC cases.

Variables	*N* (%)
Age	
Median (range)	72.5 (42–89)
Sex	
Male	64 (65.3)
Female	34 (34.7)
Histology	
Adenocarcinoma	62 (63.3)
Squamous cell carcinoma	27 (27.6)
Adenosquamous cell carcinoma	1 (1.0)
Large cell carcinoma	7 (7.1)
Carcinosarcoma	1 (1.0)
pStage	
IB	30 (30.6)
IIA	11 (11.2)
IIB	29 (29.6)
IIIA	18 (18.4)
IIIB	10 (10.2)

Abbreviation: NSCLC, non‐small cell lung cancer.

### PD‐L1 immunohistochemical staining

The PD‐L1 expression according to assay and histology is presented in Table 2. The results of PD‐L1 expressions with SP263 and 22C3 were as follows: Negative, 47 (48.0%) and 35 (34.3%) cases; weakly positive, 35 (35.7%) and 43 (43.9%) cases; and strongly positive, 16 (16.3%) and 20 (20.4%) cases, respectively. The number of negative, weakly positive, and strongly positive cases among adenocarcinoma cases were 32 (51.6%), 25 (40.3%), and five (8.1%), respectively, with SP263, and 25 (40.3%), 30 (30.6%), and seven (7.1%) with 22C3; the respective number of cases among squamous cell carcinoma cases was seven (25.9%), 10 (37.0%), and 10 (37.0%) with SP263, and three (11.1%), 12 (44.4%), and 12 (44.4%) with 22C3. Adenosquamous cell carcinoma case was strongly positive and carcinosarcoma case was negative with both assays, and one out of seven large cell carcinoma cases was weakly positive with 22C3 and negative with SP263, and all other six cases were negative with both assays (Table 2).

**TABLE 2:** Overall PD‐L1 positivity according to assay and histology.

Histology	All cases	Adenocarcinoma	Squamous cell carcinoma	Adeno‐squamous carcinoma	Large cell carcinoma	Carcino‐sarcoma
(*n*)	(98)	(62)	(27)	(1)	(7)	(1)
SP263	Cases *n* (%)					
<1%	47 (48.0)	32 (51.6)	7 (25.9)	0	7 (100)	1 (100)
1%–50%	35 (35.7)	25 (40.3)	10 (37.0)	0	0	0
≥50%	16 (16.3)	5 (8.1)	10 (37.0)	1 (100)	0	0
22C3	Cases *n* (%)					
<1%	35 (35.7)	25 (40.3)	3 (11.1)	0	6 (85.7)	1 (100)
1%–50%	43 (43.9)	30 (30.6)	12 (44.4)	0	1 (14.3)	0
≥50%	20 (20.4)	7 (7.1)	12 (44.4)	1 (100)	0	0

### Concordance of PD‐L1 expressions among assays

**FIGURE 1:**
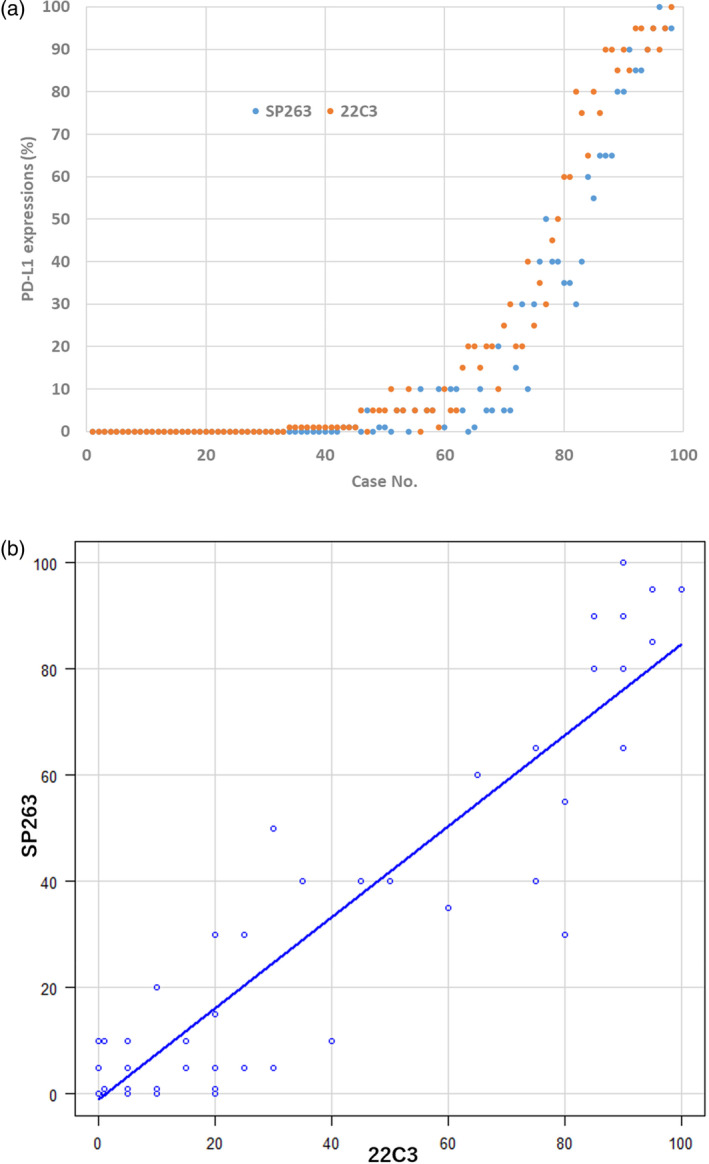
(a) Analytical comparison of programmed death ligand‐1 (PD‐L1) expression by case with each assay. (b) Scatter diagrams illustrating the correlation between two assays.

At the 1% cutoff, OPA, PPA, and NPA were 83.7%, 96.0%, and 70.2%, respectively; at the 50% cutoff, OPA PPA, and NPA were 93.9%, 93.8%, and 93.9%, respectively. The distribution of PD‐L1 expression for each assay is shown in Figure 1a. PD‐L1 expression in 22C3 was slightly higher than that in SP263 assay. The concordance between the two assays using Cohen's kappa was *κ* = 0.670 (95% CI: 0.522–0.818) at the 1% cutoff and *κ* = 0.796 (95% CI: 0.639–0.954) at the 50% cutoff (Table 3). The correlation between the two assays and the ICC was 0.937 (95% CI: 0.893–0.961), indicating high concordance. The ICC calculated separately for the retrospective group and prospective group was 0.916 (95% CI: 0.831–0.957) and 0.958 (95% CI: 0.924–0.977), respectively. Spearman's correlation coefficient was 0.874 (*p* < 0.01), indicating a strong association between the two assays (Figure 1b). Spearman's correlation coefficients for the retrospective and prospective groups were 0.863 (*p* < 0.01) and 0.879 (*p* < 0.01), respectively (Table 4).

**TABLE 3:** Agreement between the SP263 and 22C3 assays using cutoff.

Cut off	Agreement (*κ*)	OPA (%)	PPA (%)	NPA (%)
1% cutoff	0.670	83.7	96.0	70.2
50% cutoff	0.796	93.9	93.8	93.9

Abbreviations: NPA, negative percent agreement; OPA, overall percent agreement; PPA, positive percent agreement.

**TABLE 4:** Agreement between the SP263 and 22C3 assays for continuous TPS of 0%–100%.

Group	ICC	95% CI	Spearman correlation coefficient	*p*‐value
All cases	0.937	0.893–0.961	0.874	<0.01**
Retrospective group (*n* = 46)	0.916	0.831–0.957	0.863	<0.01**
Prospective group (*n* = 52)	0.958	0.924–0.977	0.879	<0.01**

Abbreviations: CI, confidence interval; ICC, intraclass correlation coefficient; TMP, tumor proportion score.

** Denotes statistical significance.

### Pathologists who evaluated PD‐L1 expressions

PD‐L1 expression with SP263 assay was evaluated by five pathologists, and 22C3 assay expression was evaluated by six pathologists, including the same five pathologists who evaluated SP263. A total of 19 cases were evaluated by the same pathologist for both assays, and 79 cases were evaluated by different pathologists. The ICC of the same pathologist group was 0.918 (95% CI: 0.776–0.969) and that of the different pathologist groups was 0.944 (95% CI: 0.905–0.966).

## DISCUSSION

In this study, the SP263 and 22C3 assays showed high concordance, with an ICC of 0.937 (95% CI: 0.893–0.961) and a Spearman correlation coefficient of 0.874 (*p* < 0.01). Using the cutoff value at the 50% cutoff, OPA, PPA, and NPA were all high, at over 90%. At the 1% cutoff, PPA was high (96%), but NPA was 70.2%. This might be because 14 patients with <1% in SP263 were scored >1% in 22C3 assay. The moderate agreement at the 1% cutoff, (*κ* = 0.67) in Cohen's kappa was affected by the cases that were negative in SP263 and positive in 22C3 assay. At the 50% cutoff, Cohen's kappa indicated strong agreement (*κ* = 0.80). Ratcliffe et al. found that the interobserver agreement was low for samples with low PD‐L1 expression (with <10%).^15^ In this study, Cohen's kappa was lower with a 1% cutoff than with a 50% cutoff. Interobserver variability may have affected these discrepancies.

There have been reports of both high concordance and discordance of PD‐L1 assays. The largest assay comparison study with 493 NSCLC biopsy samples demonstrated a high analytical correlation between SP263 and 22C3.^15^ Kim et al. reported that OPA was 91% using the 1% cutoff with 45 surgical specimens.^16^ Zhou et al. used the samples from the phase III IMpower010 study and reported that OPA was 83% using the 1% cutoff and 92% using the 50% cutoff.^17^ However, some studies have reported more positive cases with SP263 than with 22C3; they also indicated that the assays were not compatible. Hendry et al. reported that SP263 consistently classified more cases as positive than 22C3 assay.^18^ Munari et al. found that at a 50% cutoff, approximately half of the positive cases with SP263 were negative with 22C3 cases.^19^ This discordance was attributed to tumor heterogeneity, the number of tumor cells in the specimen, and variations in the performance of the staining platform and the pathologists.^20^ In this study, all cases were tested for primary lesions because discordant expression between the primary tumor and metastatic lymph nodes was reported with a kappa value of 0.546 (*p* < 0.01).^21^ In addition, there were substantial inconsistencies in PD‐L1 expressions among different tissue microarray cores in the same tumor.^22^ Hendry et al. reported that among the cases showing a positive result in one or both cores, only 43% showed a positive result in both cores.^18^ Therefore, we used the same formalin‐fixed tissue blocks for both assays to minimize the effects of intratumor heterogeneity. Moreover, PD‐L1 staining requires at least 100 tumor cells, as reported by Sughayer et al., and the discordant cases between the 22C3 and SP263 assays were all from small biopsy samples. Nevertheless, all specimens contained at least 100 tumor cells.^23^ This implies that specimens with higher cell counts, such as surgical specimens, may exhibit fewer discrepancies.

The fading of PD‐L1 expression with the age of the tumor block is known and it is especially significant when samples are older than 3 years.^16^, ^24^, ^25^ In our study, PD‐L1 expression was not tested simultaneously in both assays. Particularly in the retrospective group, 22C3 was tested later, with a time lag of up to 7 months. The immunohistochemistry procedure was exactly the same for retrospectively and prospectively collected specimens. Contrary to reports that PD‐L1 is underestimated as the age of the specimens, the results were higher for 22C3. The agreement rates calculated separately for the retrospective and prospective groups were both high, with an ICC of 0.916 (95% CI: 0.831–0.957) and 0.958 (95% CI: 0.924–0.977), respectively. A study comparing PD‐L1 expression rates by sample age showed 31.2% for <3 months, 32.8% for ≥3 months to 1 year, 29.1% for 1–3 years, and 13.3% for >3 years. The sample aged <3 years showed a higher agreement rate with the most recent sample (26). In our study, the time difference did not appear to have a significant effect on PD‐L1 expression.

Inter‐ and intraobserver variabilities often become problematic when comparing assays. Munari et al. studied 198 cases of early‐stage lung cancer and concluded that the 22C3 and SP263 assays were not interchangeable. However, interobserver agreement was moderate (*κ* = 0.72–0.77) in Cohen's kappa between two pathologists.^19^ Ratcliffe et al. compared SP263 and 22C3 in 493 NSCLC samples and demonstrated a strong analytical correlation. In their study, the variation between two pathologists scoring the same stained sample was greater than the variation between different assays scored by one reader, and they reported that assay‐specific variation was smaller than interobserver variation.^15^ In our study, the agreement rates of the same pathologist group and different pathologist groups were both high (ICC = 0.918 and 0.944, respectively); however, interobserver variability may have affected the discrepant cases. Our study detected a slightly higher frequency of positivity for 22C3 than for the SP263 assay. This result differs from previous reports, which revealed that SP263 had a higher frequency of positivity than the 22C3 assay.^10^, ^19^ Even though SP263 is the companion diagnostic for atezolizumab as adjuvant therapy, some institutes use 22C3 in its place where platforms are not available. We believed that substituting 22C3 might exclude patients who can be administered adjuvant atezolizumab. Our results mitigate this concern and have great clinical significance because it is difficult to introduce the SP263 assay platform for adjuvant atezolizumab at all institutes.

The present study had mainly three strengths. First, we focused on adjuvant therapy with atezolizumab and included only surgical specimens from complete resection cases. Second, considering the heterogeneity of PD‐L1 expression, we used the same formalin‐fixed tissue sections from the primary lesion for both assays. Third, PD‐L1 staining and scoring were outsourced to ensure objectivity. This study also had some limitations. The first limitation was the interobserver variability. Because each case was randomly evaluated by different pathologists, it could not be calculated or excluded. The second limitation was the time lag between the SP263 and 22C3 assays. Although the time lag was less than 1 year, the effect of fading cannot be completely ruled out. Third, the therapeutic effects of atezolizumab were not examined. The most important purpose was to correctly select patients who would benefit from adjuvant atezolizumab. By examining the therapeutic effects in patients whose results differed between the two assays, we can conclude whether 22C3 is a viable alternative to SP263.

In conclusion, this study showed a high concordance of PD‐L1 expression with the SP263 and 22C3 assays, with the 22C3 assay having a slightly higher frequency of positivity. These results suggest that the 22C3 assay could be used instead of the SP263 assay to determine whether to administer adjuvant atezolizumab; however, the possibility of being overestimated as a positive result with 22C3 should also be considered. Further studies examining the therapeutic effects of adjuvant atezolizumab are required.

## AUTHOR CONTRIBUTIONS

Naoko Shigeta: Data curation‐equal, formal analysis‐equal, writing – original draft‐equal. Shuji Murakami: Conceptualization‐equal, methodology‐equal, supervision‐equal, visualization‐equal, writing – review and editing‐equal. Tomoyuki Yokose: Conceptualization‐equal, methodology‐equal, resources‐equal, writing – review and editing‐equal. Tetsuya Isaka: Writing – review and editing‐equal. Kanako Shinada: Writing – review and editing‐equal. Takuya Nagashima: Writing – review and editing‐equal. Hiroyuki Adachi: Writing – review and editing‐equal. Shunsuke Shigefuku: Writing – review and editing‐equal. Kotaro Murakami: Writing – review and editing‐equal. Jun Miura: Writing – review and editing‐equal. Noritake Kikunishi: Writing – review and editing‐equal. Kozue Watabe: Writing – review and editing‐equal. Haruhiro Saito: Project administration‐equal, supervision‐equal, Writing – review and editing‐equal. Hiroyuki Ito: Project administration‐equal, supervision‐equal, writing – review and editing‐equal.

## CONFLICT OF INTEREST STATEMENT

Shuji Murakami reports personal fees from AstraZeneca, Chugai Pharmaceutical, Boehringer Ingelheim, Taiho Pharmaceutical, Ono Pharmaceutical, and Riken Genesis. Terufumi Kato reports grants and personal fees from MSD, Novartis, Ono Pharmaceutical, Pfizer, and Taiho Pharmaceutical, personal fees from Daiichi Sankyo, F. Hoffmann‐La Roche, Nippon Kayaku, Nitto Denko, Shionogi Pharmaceutical, Sumitomo Dainippon, and Takeda, and grants from Astellas, Kyorin, Kyowa Kirin, and Regeneron. Haruhiro Saito reports grants from Chugai Pharmaceutical and AstraZeneca, and personal fees from Ono Pharmaceutical, Nippon Boehringer Ingelheim, MSD, and Novartis Pharma.

## Data Availability

The data that support the findings of this study are available on request from the corresponding author (M.S.).
